# Factors associated with caregiver compliance to an HIV disclosure intervention and its effect on HIV and mental health outcomes among children living with HIV: *post-hoc* instrumental variable-based analysis of a cluster randomized trial in Eldoret, Kenya

**DOI:** 10.3389/fpubh.2023.1150744

**Published:** 2023-05-05

**Authors:** Elizabeth B. Magill, Winstone Nyandiko, Aaron Baum, Josephine Aluoch, Ashley Chory, Celestine Ashimoshi, Janet Lidweye, Tabitha Njoroge, Festus Sang, Jack Nyagaya, Michael Scanlon, Joseph Hogan, Rachel Vreeman

**Affiliations:** ^1^Department of Health Systems Design and Global Health, Arnhold Institute for Global Health, Icahn School of Medicine at Mount Sinai, New York, NY, United States; ^2^Academic Model Providing Access to Healthcare (AMPATH), Eldoret, Kenya; ^3^Department of Child Health and Pediatrics, School of Medicine, College of Health Sciences, Moi University, Eldoret, Kenya; ^4^Center for Global Health, Indiana School of Medicine, Bloomington, IN, United States; ^5^Department of Biostatistics, Brown University School of Public Health, Providence, RI, United States

**Keywords:** mental health, Kenya, pediatric HIV, HIV status disclosure, caregiver intervention

## Abstract

**Background:**

The HADITHI study is a cluster-randomized trial of children living with HIV and their caregivers in Kenya that aimed to increase rates of caregiver disclosure of their child's HIV status, encourage earlier status disclosure, and improve pediatric mental health and HIV outcomes. This analysis identified characteristics predicting caregiver non-responsiveness and compared outcomes among children based on disclosure status.

**Methods:**

A penalized logistic regression model with lasso regularization identified the most important predictors of disclosure. The two-stage least squares instrumental variable approach was used to assess outcomes accounting for non-compliance to disclosure.

**Results:**

Caregiver non-isolation and shorter time on antiretroviral therapy were predictive of HIV status disclosure. There were no statistically significant differences found in CD4 percentage, depression status, or mental and emotional status based on disclosure status up to 24 months-post intervention.

**Conclusion:**

These findings have implications for specialists seeking to tailor disclosure interventions to improve caregiver-child dyad responsiveness.

## Introduction

In 2021, there were approximately 1.7 million children living with HIV and 160,000 children newly infected with HIV globally (1). As these children age, appropriate timing and methods of disclosure of HIV status become important components in the management of their health. The World Health Organization recommends that the HIV disclosure process for children perinatally infected with HIV should be started by age six and completed by age twelve (2). Adolescents living with HIV who are aware of their HIV status have been found to have improved adherence to antiretroviral treatment (3–8), knowledge of sexual and reproductive health (5, 8), and psychological wellbeing (3, 5, 9) across multiple global settings. In addition, disclosure provides adolescents critical autonomy and personal control over their health, which becomes increasingly important as they navigate the lifelong impact of their HIV status (10, 11).

Despite the clear importance of disclosure for children living with HIV, most adolescents in resource-limited settings remain undisclosed. Up to 50% of adolescents across studies in low and middle-income countries were told non-HIV related reasons for HIV illness and healthcare visits (12). These differences are most prominent in African countries, which are home to 75% of children living with HIV under the age of fifteen and have HIV disclosure rates for HIV-positive adolescents between 15 and 64% (4, 13–17). Qualitative studies have found that caregivers choose not to disclose their child's HIV positive status with them due to their beliefs about HIV-related stigma, including stereotypes associating HIV with immorality and death, concerns about the impact of others' stereotypes about HIV on the child, worry about how others will treat their family if others found out about the diagnosis, and fear of a negative psychological reaction from the child (18–21).

Interventions focused on HIV disclosure have been developed across the world—including in Kenya (22, 23), Ghana (24), Namibia (25, 26), Puerto Rico (27), Uganda (28), and the United States (29)—to support caregivers in disclosing HIV status to their children. Overall, these interventions have been found to increase prevalence of HIV disclosure (22, 24, 25) and improve HIV treatment adherence (25, 27) with normalized or improved mental health within 1 year post-intervention (22, 27–29). In multiple settings, youth and caregivers considered disclosure a positive event for their families (26, 27).

Based on this literature, it is well-understood that HIV disclosure interventions are successful in improving disclosure rates and play an important role in supporting the overall health and wellbeing of adolescents living with HIV. Yet, an important assumption remains unquestioned from existing research: is the action of disclosure itself, or participation in a disclosure intervention, more predictive of improved health outcomes? In other words, does disclosure matter after a dedicated HIV disclosure intervention, or could these same outcomes be found from participation in the intervention even without disclosure? Relatedly, are there common characteristics among caregivers who decide to disclose post-intervention, and are these relevant to their child's post-disclosure outcomes?

This study attempts to answer these questions by employing instrumental variable analysis to isolate disclosure as an independent variable separate from intervention participation using data from a cluster-randomized controlled trial of an HIV disclosure intervention conducted in Kenya from 2013–2015 (22). Using this statistical methodology, we analyze factors associated with compliance to disclosure after completion of a disclosure intervention and assess the impact of disclosure on HIV, mental, and behavioral health outcomes of adolescents living with HIV.

## Methods

### The HADITHI intervention for HIV disclosure

The Academic Model Providing Access to Healthcare (AMPATH) Consortium is a partnership established in 2001 between 14 universities and academic health centers across North America and Moi University and Moi Teaching and Referral Hospital in Eldoret, Kenya that aims to provide comprehensive and preventative care, advance research findings, and educate medical students, residents, and community healthcare workers. Based on over a decade of medical practice and research related to pediatric HIV/AIDS in western Kenya, the AMPATH consortium developed a culturally adapted, multicomponent intervention to support disclosure of HIV status to perinatally infected children referred to cumulatively as the HADITHI (“Helping AMPATH Disclose Information and Talk about HIV Infection”) intervention. The disclosure intervention included patient-centered materials to guide disclosure, disclosure counselors, and post-disclosure child support groups to supplement usual care resources (30).

A cluster randomized controlled trial (Vreeman 1R01MH0099747-01, “Patient-Centered Disclosure Intervention for HIV-Infected Children”) conducted between 2013 and 2015 evaluated the effectiveness of the HADITHI intervention on 285 caregiver-child dyads recruited from eight facilities in Eldoret, Kenya using an as-treated approach with intensive clinical and psychosocial assessments at 6-month intervals until 2 years post-intervention. The primary outcome was prevalence of HIV disclosure and pre-specified secondary outcomes included mental and behavioral outcomes for children living with HIV. The study was approved by the Institutional Review Board at Indiana University School of Medicine in Indianapolis, Indiana, USA and by the Institutional Research Ethics Committee at Moi University School of Medicine in Eldoret, Kenya. Additional design and results of the primary HADITHI study have been previously reported (22).

### Current study design

The overall objective of this *post-hoc* study was to examine the effect of non-adherence to disclosure among HADITHI intervention participants. In this study, we applied instrumental variable estimation to the intention-to-treat analysis previously published in 2019 (22). Our primary aims were 2-fold: (1) identify covariates that predict caregiver responsiveness to the HIV disclosure HADITHI counseling intervention and (2) estimate the local average treatment effect of disclosure on child HIV and mental health outcomes, including CD4 percentage, child depression, and child mental and behavioral status.

### Patient selection

Two hundred and eighty five caregiver-child dyads were recruited for the HADITHI trial from eight facilities in Eldoret, Kenya between June and August 2013. We restricted our analysis to children who were not disclosed to at baseline. Disclosure was defined as a binary variable (whether or not the child knew his/her HIV status) as reported by either child or caregiver via disclosure questionnaires. Of the 285 caregiver-child dyads, 146 children (51.2%) were non-disclosed at baseline by either caregiver or child report.

### Measures

Disclosure post-intervention was defined as a binary variable of whether or not the child knew his/her HIV status as reported by both child and caregiver via disclosure questionnaires. Disclosure was assessed at 6-month intervals from baseline (immediately post-intervention) to 24 months post-intervention.

Eighty demographic and clinical covariates were extracted from children's medical files or compiled from baseline questionnaires provided to caregivers and children during the HADITHI trial, including the Pediatric AIDS Clinical Trials Group General Health Assessment for Children Quality of Life Questionnaire, Strengths and Difficulties Questionnaire- Youth Version (SDQ), Patient Health Questionnaire nine-item depression instrument (PHQ-9), and locally developed and validated adherence and Stigma in AIDS family adherence (SAFI) stigma questionnaires (Supplementary Table 1). Details regarding measures, their administration, and their use in the HADITHI trial has been previously published (31, 32).

Outcome measurements for instrumental variable analysis were assessed using data from post-intervention follow-up at 6 month intervals for 24 months. CD4 percentage was extracted from the children's medical files by trained study team members. Depression symptoms were measured by the PHQ-9, and due to low frequencies of children reporting depression, the scales were transformed into three severity categories: no depression (score of 0), minimal symptoms (score of 1–4), and clinically significant depression (score of 5–19). Overall emotional and behavioral symptoms were measured by the SDQ, and scores were categorized into three categories: normal (score of 0–15), borderline (score of 16–19) and abnormal (score of 20–40).

### Statistical analysis

Sample characteristics and distributions of categorical predictors were summarized using numbers and percentages for categorical variables, mean and standard deviation for normalized continuous variables, and median and interquartile ranges for non-normalized continuous variables. Baseline characteristics of children in the intervention and control groups were compared using Pearson chi-square tests, Fisher's exact tests, two-sample *t* tests, and two-sample Wilcoxon rank sum tests, as appropriate to test for statistical significance.

#### Caregiver compliance to disclosure

The least absolute shrinkage and selection operator (LASSO) penalized regression was used to select the best subset of predictors of disclosure for the 60 caregiver-child dyads who were randomized into the intervention group. We defined disclosure as a binary response of “disclosed” vs. “not disclosed,” with disclosure defined as caregiver-reported disclosure at any point within the 24-month follow-up period. Caregiver-child dyads who participated in the intervention were analyzed to select the best subset of multi-level predictors (HIV, mental health, overall health, economic, household, or community-related factors) of disclosure. LASSO selects a subset of predictors by shrinking the coefficients of the least contributive variables to zero, thereby excluding them from the model. LASSO combines the benefits of multiple regularizations and is particularly useful in studies such as this one where the number of observations is less than the number of variables and there are groups of correlated variables.

To identify relevant predictors, all explanatory variables available in the data set were entered into the LASSO procedure. To calculate a reliable measure for the model validity, we used the conventional validation technique and randomly split the data into two data sets: 50% as the training data on which variable selection via the LASSO was done and 50% as the test data on which the logistic regression and corresponding pseudo R-squared were calculated. The dependent variable was disclosure after baseline. Independent variables included 80 demographic and clinical covariates described above. In the testing data, the most important predictors were selected using validation alpha standard error (ASE). To reduce the random effect arising out of the random split of the data, we used the bootstrapping method to repeat the random partition 1,000 times with replacement, and the average value of the obtained ASEs was calculated to produce the best regularization model (33).

#### Local average treatment effect

**Figure 1:**
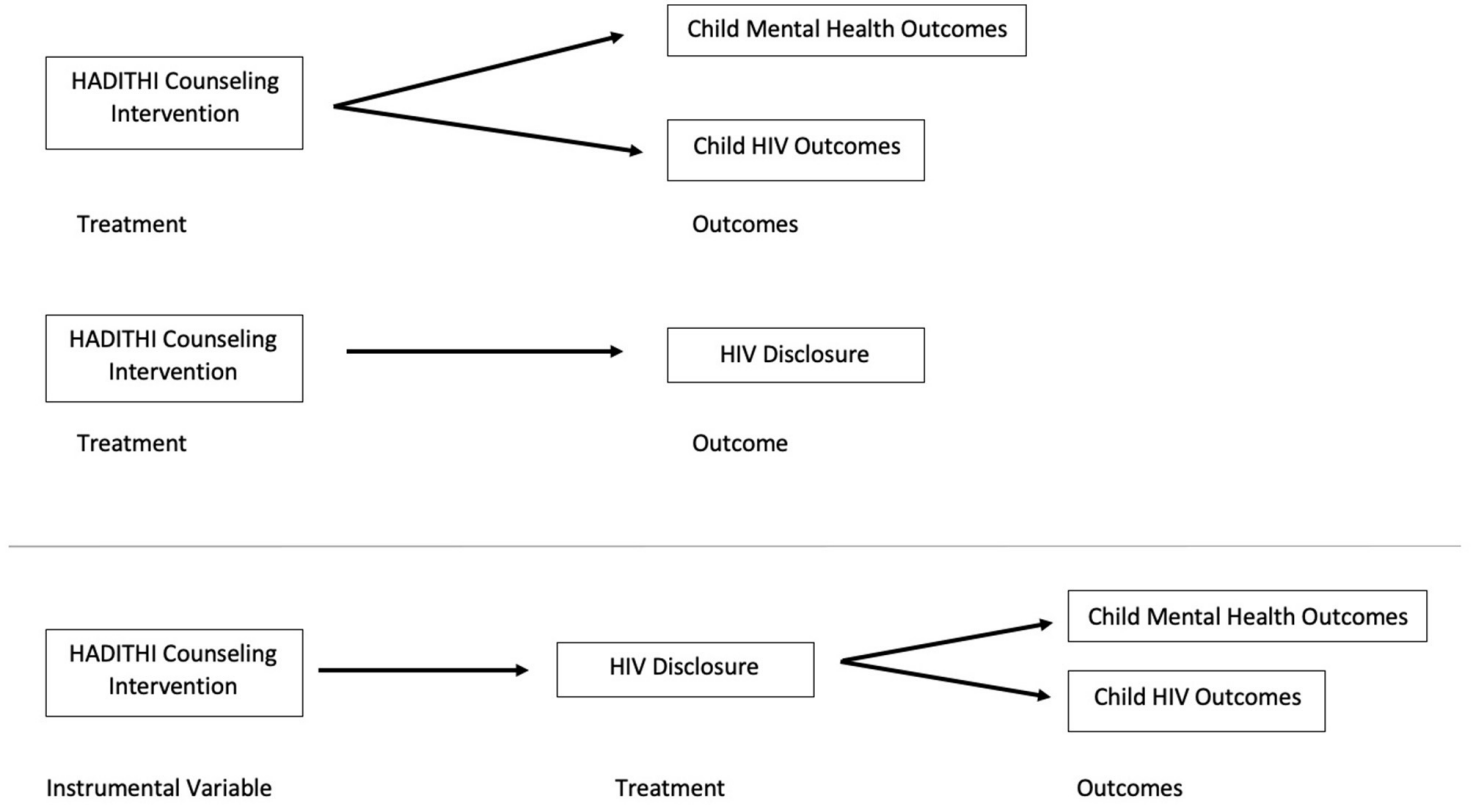
Causal diagram for intent-to-treat HADITHI intervention data analysis with comparison updated casual diagram for instrumental variable analysis isolating HADITHI counseling intervention from HIV disclosure to depict potential non-compliance.

Instrumental variable (IV) methods were used to assess outcomes of children with HIV to account for non-compliance to disclosure among intervention participants (Figure 1). While standard intent-to-treat analysis estimates the effect of treatment assignment on outcomes, this does not carry causal interpretation in the presence of treatment non-compliance. IV methods provide an alternative approach by using “instruments” to isolate the variance in outcome that is due to non-compliance of the intervention (34, 35). IV analysis was chosen because its estimates have been shown to be unbiased when non-compliant behaviors are symmetrically dependent on patients' conditions (36), which was consistent with other studies of HIV disclosure compliance (4, 14, 16).

Randomization at the clinic level was used as the instrument for IV analysis because this instrument satisfied the two required assumptions: (1) the instrument affects the processes patients receive and (2) the instrument is not correlated with unmeasured factors or directly related with outcomes (37, 38). Randomization into the intervention or control group directly affected the treatment that the patient received. It was expected that the cluster randomization and the study designs would also not be correlated with unmeasured factors affecting study outcomes. However, this may not always be the case since cluster randomization at a clinic level may not fully balance patient characteristics between groups. When randomization influences treatment monotonically, IV methods estimate the causal effect among the adherers, also known as the local average treatment effect (LATE) or complier-average causal effect (39).

IV models were estimated using the two-stage least squares (2SLS) approach (40). The fully specified 2SLS model for each outcome model included a first-stage disclosure equation that was explained by the control variables (gender, age, and tribe) and one indicator variable of the instrument (variable = 1 if the subject was in the intervention group, otherwise 0). Because the outcome measures were repeated measures, the model also included a variable representing time since disclosure and an interaction term of time period^*^intervention status. In the second stage of 2SLS, the predicted number of disclosures and the same set of control variables were used to estimate the process effects on the outcome. The second stage 2SLS model was run independently for data at post-intervention time points of 6, 12, 18, and 24 months. The regression coefficient for the predicted treatment received in the second stage of the model is a consistent estimator of the LATE if the first stage model is a linear regression containing all the variables appearing in the second stage (41, 42).

As a comparison, ordinary-least-squares (OLS) linear regression models were utilized to estimate the effects of the processes on outcomes using an as-treated approach. The outcome model was explained by intervention status and the control variables again run independently at all four post-intervention time points.

SAS Studio 3.8 (Enterprise Edition) was used in managing data, performing descriptive statistics, comparisons, and diagnostic tests, and completing the regression analysis. A statistical significance level of 5% was utilized for all analyses, and clinical significance was defined as a 5-fold change in the test statistic.

## Results

### Baseline demographic and clinical characteristics

Of the 146 non-disclosed caregiver-child dyads who participated in the HADITHI intervention, 130 (89%) completed all follow-up assessments and were included in this secondary analysis.

Among all non-disclosed participants, the median child age was 11.42 years old and 55% were girls (Table 1). The majority of primary caregivers (*n* = 86) were the child's biological mother. Forty eight children (37.5%) had Stage 3 HIV disease at baseline, while 37 children (28.9%) had Stage 2 disease and 39 children (30.5%) had Stage 1 disease. Eighty-five percent of children were on ART at the initiation of the intervention. Forty-five percent of children had minimal depressive symptoms below clinical levels of depression, and 20% of children had clinically mild to moderately severe depression.

**Table 1:** Baseline demographics and clinical characteristics.

			**Total non–disclosed**	**Control subset**	**Intervention subset**	
**Variable**		***n***	**Mean ±SD or median (IQR) or *n* (%) (*n =* 130)**	**Mean ±SD or median (IQR) or *n* (%) (*n =* 70)**	**Mean ±SD or median (IQR) or *n* (%) (*n =* 60)**	***P***
Age (years)		130	11.42 (9.60–13.24)	11.56 (9.42–13.70)	11.33 (9.73–12.93)	0.2643^d^
Male		130	59 (45.38%)	28 (40.00%)	31 (51.67%)	0.1829^a^
Attend school		128	127 (99.22%)	68 (98.55%)	59 (100%)	1.000^b^
Parent dead (at least one)		130	60 (46.15%)	36 (51.43%)	24 (40.00%)	0.1926^a^
Orphaned (both parents dead)		130	24 (18.46%)	16 (22.86%)	8 (13.33%)	0.1629^a^
Sibling with HIV		121	23 (19.01%)	14 (21.21%)	9 (16.36%)	0.4985^a^
WHO Clinical Stage		128				0.3384^b^
	1		39 (30.47%)	24 (35.29%)	15 (25.00%)	
	2		37 (28.91%)	21 (30.88%)	16 (26.67%)	
	3		48 (37.50%)	22 (32.35%)	26 (43.33%)	
	4		4 (3.13%)	1 (1.47%)	3 (5.00%)	
Tribe		129				< 0.0001^b^
	Kalenjin		34 (26.36%)	10 (14.49%)	24 (40.00%)	
	Kikuyu		22 (17.05%)	5 (7.25%)	17 (28.33%)	
	Luhya		43 (33.33%)	31 (44.93%)	12 (20.00%)	
	Luo		25 (19.38%)	20 (28.99%)	5 (8.33%)	
	Other		5 (3.88%)	3 (4.35%)	2 (3.33%)	
On ART		130	110 (84.62%)	57 (81.43%)	53 (88.33%)	0.2767^a^
Duration on ART (years)		119	4.37 ± 2.36	4.12 ± 2.29	4.67 ± 2.42	0.2040^c^
Regimen		83				0.4653^b^
	First Line		75 (90.36%)	41 (93.18%)	34 (87.18%)	
	Second Line		8 (9.64%)	3 (6.82%)	5 (12.82%)	
CD4+ percentage		115	27.0 (16.0–38.0)	29.0 (18.0–40.0)	26.0 (14.0–38.0)	0.2206^d^
BMI–for–age Z scores		92	−0.77 ± 1.11	−0.66 ± 1.08	−0.90 ± 1.13	0.3061^c^
Height–for–age Z scores		92	−1.55 ± 1.32	−1.39 ± 1.37	−1.72 ± 1.25	0.2397^c^
PHQ−9 scores		130				0.4240^a^
	No depressive symptoms		44 (33.85%)	23 (32.86%)	21 (35.00%)	
	Minimal depressive symptoms		59 (45.38%)	35 (50.00%)	24 (40.00%)	
	Mild to moderately severe depression		27 (20.77%)	12 (17.14%)	15 (25.00%)	
SDQ scores		130				0.1280^a^
	Normal		100 (68.49%)	50 (71.43%)	39 (65.00%)	
	Borderline		29 (19.86%)	16 (22.86%)	11 (18.33%)	
	Abnormal		17 (11.64%)	4 (5.71%)	10 (16.67%)	
Caregiver		130				
	Mother		86 (58.90%)	41 (58.57%)	34 (56.67%)	0.9522^a^
	Father		24 (18.46%)	13 (18.57%)	11 (18.33%)	0.9722^a^
	Sibling		7 (5.38%)	3 (4.29%)	4 (6.67%)	0.7030^b^
	Grandparent		16 (12.31%)	10 (14.29%)	6 (10.00%)	0.4584^a^
	Aunt/Uncle		25 (19.23%)	12 (17.14%)	13 (21.67%)	0.5141^a^
	Other		5 (3.85%)	4 (5.71%)	1 (1.67%)	0.3728^b^

ART, antiretroviral therapy. ^a^Pearson's Chi-square test. ^b^Fisher's exact test. ^c^Two-sample *t*-test. ^d^Two-sample Wilcoxon rank-sum test.

Demographic characteristics were not significantly different between non-disclosed caregiver-child dyads in the control and intervention subgroups except for tribe (*p* < 0.0001). The plurality of non-disclosed dyads in the control were members of the Luhya tribe (45%), while the plurality of non-disclosed dyads in the intervention were members of the Kalenjin tribe (40%).

### Caregiver compliance to disclosure

Ninety-one percent of caregiver-child dyads who participated in the HADITHI intervention completed follow-up disclosure questionnaires until 24 months post-intervention. Eighty clinical and demographic covariates collected from the 60 caregiver-child dyads who participated in the HADITHI intervention were entered into the regression procedure. Two of the 80 predictors—caregiver isolation (test statistic = 8.79, *p* = 0.0030) and length of time on ARVs (test statistic = 5.20, *p* = 0.0226)—were retained as significant in the regression model (Table 2). The model's discrimination between caregivers' disclosure status was strong, with an area under the curve (AUC) = 81.21%.

**Table 2:** Child and caregiver characteristics predicting post-intervention HIV disclosure status.

	**Disclosure**	**Non-disclosure**	**Wald chi-square**	**Pr > ChiSq**	**Odds ratio**	**95% CI**
Years on ART (SD)	1.3 (1.5)	2.5 (2.4)	5.2	0.02	0.99	0.99, 0.99
Caregiver isolation (+)	3	5	8.78	0.01	13.13	2.39, 72.09
Caregiver isolation (–)	44	8				

Caregivers who experienced isolation, defined as responding “ever happened” to the prompt “Because the child or someone else in my family has HIV or because I have HIV, I am isolated or avoided by others” on the SAFI stigma questionnaire, were significantly less likely to disclose their child's HIV status to them post-intervention. While 44 out of 52 (84.6%) of non-isolated caregivers disclosed HIV status after the intervention, only 3 out of 8 (37.5%) of isolated caregivers disclosed. In addition, children who had been on ARVs for an increased time prior to the intervention were less likely to be disclosed to about their HIV status post-intervention. The mean (SD) length of time on ARVs for children who were disclosed to was 1.3 (1.5) years, while that of those who were not disclosed to was 2.5 (2.4) years.

### Local average treatment effect on clinical HIV and mental and behavioral health outcomes

IV regression was conducted to compare clinical outcomes between intervention participants who disclosed HIV status post-intervention and those who did not. There were no significant differences in CD4 count, PHQ-9 scores, or SDQ scores between the children of intervention compliers and non-compliers at 6 months, 12 months, 18 months, or 24 months post-intervention (Table 3). All *p*-values were >0.16, indicating no statistical significance.

**Table 3:** Effect of intervention and disclosure on mental and psychosocial outcomes up to 24 months post-intervention.

	**6 Months**				**12 Months**				**18 Months**				**24 Months**			
	**Intervention**	***p-*** **value**	**Disclosure**	***p-*** **value**	**Intervention**	***p-*** **value**	**Disclosure**	***p-*** **value**	**Intervention**	***p-*** **value**	**Disclosure**	***p-*** **value**	**Intervention**	***p-*** **value**	**Disclosure**	**p-value**
**CD4 percentage**	−0.273	0.739	0.161	0.774	1.761	0.142	6.941	0.224	0.558	0.814	2.408	0.605	0.018	0.989	0.06	0.989
*95% confidence interval (CI)*	(−2.134, 1.588)		(−0.938, 1.260)		(−0.756, 4.278)		(−4.241, 18.122)		(−5.000, 6.114)		(−6.720, 11.536)		(−3.160, 3.195)		(−8.791, 8.910)	
**PHQ−9 score**	0.126	0.407	0.577	0.309	0.106	0.567	0.872	0.489	−0.199	0.124	−0.963	0.161	0.019	0.932	0.091	0.866
*95% CI*	(−0.212, 0.465)		(−0.536, 1.690)		(−0.312, 0.525)		(−1.598, 3.343)		(−0.469, 0.070)		(−2.312, 0.385)		(−0.484, 0.522)		(−0.964, 1.146)	
**SDL score**	0.031	0.726	−1.524	0.747	−0.042	0.799	−0.348	0.754	−0.117	0.453	−0.559	0.339	0.048	0.755	0.245	0.591
*95% CI*	(−0.170, 0.231)		(−10.773, 7.724)		(−0.420, 0.336)		(−2.523, 1.826)		(−0.464, 0.231)		(−1.706, 0.588)		(−0.300, 0.396)		(−0.649, 1.140)	

OLS regression was also conducted for comparison using the predetermined subset of non-disclosed caregiver-child dyads at baseline. There were also no significant differences in CD4 count, PHQ-9 scores, or SDQ scores between those who completed the intervention vs. those who were in the control group at any point post-intervention. All *p*-values were >0.12.

When comparing OLS and IV methodology, a clinically significant difference between models was defined as a 5-fold difference in the test statistic, or the difference in outcomes between intervention participation (OLS methodology) and disclosure (IV methodology). IV analysis found a clinically significant decrease in SDQ scores at 6 months (0.031 vs. −1.524, 49.2-fold difference) and 12 months (−0.042 vs. −0.348, 8.3-fold difference) as compared to OLS analysis, indicating improved behavioral health over the 1^st^ year for adolescents who were post-disclosure as compared to those who were post-intervention. At the same time, IV analysis also found a clinically significant increase in PHQ-9 scores at 12 months (0.872 vs. 0.106, 8.2-fold difference) and in SDQ scores at 24 months (0.048 vs. 0.245, 5.1-fold difference) as compared to OLS analysis, indicating worsened depression and behavioral health for post-disclosure adolescents as compared to post-intervention adolescents at 1 and 2 years post-intervention.

## Discussion

This study defined caregiver-child characteristics that predicted caregiver compliance to a disclosure intervention for children living with HIV and their families in western Kenya. We also used instrumental variable methods to explore local average treatment effects of disclosure post-intervention on HIV, mental health, and behavioral health outcomes. In completing this analysis, we aimed to isolate disclosure as a variable separate from intervention participation to independently assess the independent prediction and impact of disclosure.

Two caregiver-child characteristics were found to be predictive of compliance to the HIV disclosure intervention: caregiver isolation and the length of time that the child had been taking ARVs.

While almost 85% of non-isolated caregivers disclosed their child's HIV status to them after the intervention, less than 40% of caregivers who self-reported feeling isolated complied with HIV disclosure. This finding substantiates qualitative research conducted within this population prior to the intervention when caregivers cited their own isolation and fears of their child's isolation post-HIV status disclosure as reasons for not previously disclosing their child's HIV status (18). It also links to studies in other settings that cite caregiver isolation as a barrier to HIV disclosure for children living with HIV (43–45). This study used the SAFI questionnaire definition of caregiver isolation as self-report of anxiety or isolation based on their own or a family member's HIV status. This introduces additional considerations that may impact caregiver isolation leading to non-disclosure, including beliefs about HIV-related stigma and stigma regarding their own HIV status. This link has been studied in other settings, finding lack of knowledge about HIV and HIV stigma as barriers to disclosure among caregivers of children with HIV (13, 15, 16). Studies also show statistically increased likelihood of disclosure with caregiver beliefs that disclosure had benefits (14, 16) and participation in HIV-positive communities (46). Those with more isolation may not feel they have a safe place to share their worries about disclosure, and they may not have the contacts to hear about the potential benefits of disclosure. Each of these factors may contribute to the feelings of isolation self-reported by caregivers in this study.

In combination, this research suggests that HIV-related stigma may isolate caregivers and impact their willingness to disclose HIV status, even after specific curriculum within a disclosure intervention to address and attempt to combat HIV stigma. These isolated caregivers may continue to have substantial fear about the potential for a disclosed child to share about HIV status with others and subsequently be subject to further stigma. It also proposes the need for increased focus on caregiver HIV status and stigma within pediatric HIV disclosure interventions, which has already been implemented in various disclosure models (47, 48).

Caregivers of children who were on ARVs for a longer time were also less likely to disclose post-intervention in this study. The average length of time on ARVs for children who were disclosed was more than 1 year less than those who were not disclosed. This result is surprising, since other studies have found that increased length of time on ARV medication was significantly associated with HIV disclosure (14, 15, 17). This variable may substitute the length of time since a child's HIV diagnosis and may suggest that the longer a caregiver has hidden HIV status from their child and created false narratives about their HIV treatment, it may be more difficult for the caregiver to disclose to their child. One study in Zimbabwe provided insight into this potential issue, as caregivers noted that they did not disclose to their children because they felt that the child would reject the caregiver in anger for not disclosing sooner (49). This has also been found in other studies from Kenya among adults with HIV, who were shown in some studies to be less likely to disclose their own HIV status to others if they have hidden their status for a longer time (50, 51). Additional studies should assess the impact of length of time on ARVs on rates of pediatric HIV disclosure across contexts to assess when to administer disclosure interventions for optimal compliance.

Of note, this study did not find that any of the other 78 baseline caregiver-child characteristics were significantly associated with disclosure post-intervention The full list of baseline characteristics included in the regression model can be found in the Supplementary Table. Other studies have found a variety of other characteristics to be statistically significantly associated with disclosure in other settings, including: child age, place of follow-up, caregiver educational level, child weight, and child sex (4, 13–17). It is possible that a larger sample size may have found more associations, or that something specific to each study setting led to these differences. It is also important to consider that this study assesses characteristics of compliance to disclosure status post-disclosure intervention, rather than assessing prevalence of disclosure outside of an intervention. We did find similar characteristics to other studies when predicting baseline disclosure as described elsewhere (6), but only children who were not disclosed to at baseline were included in our analysis. Our study is the first to assess characteristics of compliance to disclosure after a specific intervention in this context.

Our study did not find statistically significant differences in CD4 percentage, PHQ-9 score, or SDQ score between children based on disclosure status across the intervention. Disclosure was shown to not impact mental health or HIV outcomes, which contradicts caregiver fears that disclosing HIV status would worsen children's mental health and control over HIV treatment (18), a reported reason for non-disclosure in other low-resource settings (52). These results may be encouraging to share with community members in future interventions to lessen anxieties about the potential negative impacts of disclosure for their children. In addition, there appear to be mixed clinically significant differences in PHQ-9 and SDQ scores across time points when using IV analysis as compared to OLS. Our results may suggest clinically relevant mental health improvement for adolescents with newly disclosed HIV, with improved behavioral health outcomes found at 6 and 12 months from the IV analysis, followed by clinically relevant worsening depression and behavioral health outcomes at 12 and 24 months from IV analysis. Given the lack of overall statistical significance in combination with the mixed results between the IV analysis and the OLS analysis, we cannot conclude whether disclosure impacted the mental health of adolescents who participated in the HADITHI intervention. A larger trial may show transient yet statistically significant changes in mental and behavioral health outcomes within 1 year post-disclosure that return to baseline over time.

These outcomes fit within growing literature that suggests that disclosure of HIV status to children in resource-limited settings may lead to improved HIV, psychological, and quality of life outcomes. One unmatched case control study of 309 children living with HIV in Tanzania found that patients who had their HIV status disclosed to them were more likely to have improved ART adherence as measured by a treatment adherence manual and improved quality of life as measured by World Health Organization Quality of Life standard tool (16). A second observational prospective cohort study on 160 children with HIV in Bangkok, Thailand evaluated the psychosocial outcomes of disclosure as measured by the Child Behavioral Checklist (53). Researchers found that the median depression score decreased significantly at 2-month and 6-month follow-up. Similar observational and cohort-based research has been published in Ghana (54), Namibia (25), and South Africa (55). Despite this initial evidence, however, randomized controlled trials have only assessed parental disclosure of their own HIV status to seronegative children (56, 57) rather than caregiver disclosure of a child's own HIV status. This study was the first to assess the direct impact of disclosure using a cluster-randomized control trial.

Although the HADITHI trial found improved outcomes among children living with HIV when comparing children who completed the disclosure intervention to those who did not (22), these results did not translate to this study's analysis isolating disclosure status from participation in the intervention. This suggests that participation in the intervention may support health outcomes even without ultimate compliance to disclosure. Future studies should continue to distinguish between outcomes attributed specifically to disclosure as compared to those correlated with completion of a disclosure-focused intervention.

Our study contained numerous limitations. First, this analysis was limited by its sample size since the study was not sufficiently powered for effect sizes. Almost 50% of children who participated in the HADITHI intervention were already aware of their HIV status at baseline, significantly limiting the number of caregiver-child pairs in this analysis and thus the study's power. Second, the definition of disclosure used in this quantitative analysis could not capture the nuances of disclosure studied in this and other settings. Some caregiver-child dyads reported divergent answers for disclosure across the study—with the caregiver stating that they disclosed to the child but the child not expressing knowledge of their HIV status or vice-versa. For this analysis, we defined disclosure as both the parent and the child reporting disclosure, but this may underestimate study results. In addition, it is well-studied that disclosure is not a binary variable but a longitudinal process (49, 55), which could not be addressed within the scope of this paper. Finally, the statistical methodology of instrumental variable analysis requires an assumption that the disclosure intervention itself did not impact HIV and mental health outcomes, instead attributing these changes only to disclosure. In reality, it is possible that participating in the intervention, even without disclosure, may have impacted mental health or HIV outcomes of adolescents involved in the study. This is important to note especially when comparing IV and OLS analysis, as these methods analyze different populations; IV analysis only compares adolescents who completed in the intervention based on disclosure status, while OLS analysis also includes adolescents who did not complete the intervention. This limits comparison of local average treatment effect test statistics from IV analysis and average treatment effect test statistics from OLS analysis. Despite these potential limitations, we chose the instrumental variable method to most closely approximate the isolated impact of disclosure in our study population.

## Conclusion

Our study found that caregiver isolation status and the length of time a child had been on antiretroviral therapy were predictive of disclosure of HIV status to children living with HIV in western Kenya after participation in a disclosure intervention. We also found that children who had their HIV status disclosed to them and those who did not had no statistically significant different outcomes in CD4 percentage, depression status, or mental and emotional status up to 24 months post-intervention. The results of this study can inform future adaptation of the HADITHI intervention as well as disclosure interventions in other low-resource contexts. Disclosure interventions should integrate additional mental health and treatment adherence counseling aimed at continuing to stabilize mental health post-disclosure and improving mental health and HIV outcomes over time. Educational components of disclosure interventions can also be tailored to include concepts related to HIV-related stigma and caregiver and family isolation and target new ARV patients. Future research should replicate this study design elsewhere to understand factors contributing to disclosure compliance in other settings and further explore the impact of disclosure on health outcomes at further timepoints post-disclosure.

## Data availability statement

The original contributions presented in this study are included in the article/Supplementary material. Additional data from the original HADITHI study and further inquiries can be directed to the corresponding author.

## Ethics statement

The studies involving human participants were reviewed and approved by Institutional Review Board at Indiana University School of Medicine in Indianapolis, Indiana, USA, and Institutional Research Ethics Committee at Moi University School of Medicine in Eldoret, Kenya. Written informed consent to participate in this study was provided by the participants' legal guardian/next of kin.

## Author contributions

EM, AB, and RV designed the research. WN, JA, CA, JL, TN, FS, JN, MS, and JH proved substantial contributions to the conception of the project and the acquisition of project data. EM completed data analysis and manuscript drafting. WN, AB, AC, and RV contributed to manuscript revision. All authors contributed to the article and approved the submitted version.
